# Post-Mortem Tissue Biopsies Obtained at Minimally Invasive Autopsy: An RNA-Quality Analysis

**DOI:** 10.1371/journal.pone.0115675

**Published:** 2014-12-22

**Authors:** Anita van der Linden, Britt M. Blokker, Marcel Kap, Annick C. Weustink, Peter H. J. Riegman, J. Wolter Oosterhuis

**Affiliations:** 1 Department of Pathology, Erasmus MC, Rotterdam, The Netherlands; 2 Department of Radiology, Erasmus MC, Rotterdam, The Netherlands; University of Palermo, Italy

## Abstract

**Introduction:**

Bereaved relatives often refuse to give consent for post-mortem investigation of deceased cancer patients, mainly because of the mutilation due to conventional autopsy (CA). Minimally invasive autopsy (MIA) may be a more acceptable alternative and, if implemented in clinical practice, creates an opportunity to more often obtain post-mortem tissue samples of (recurred) primary tumors and metastases for molecular research. As a measure for tissue quality for molecular studies, we hereby present a feasibility study, comparing the RNA quality of MIA and CA samples, and fresh frozen samples as reference.

**Materials and methods:**

Tissue samples of heart, liver and kidney were prospectively collected from 24 MIAs followed by CA, and compared to corresponding archival fresh frozen tissue. After RNA isolation and RT-qPCR, RNA integrity numbers (RIN) and *GAPDH* expression (six amplicon sizes ranging from 71 to 530 base pairs) were measured. RIN values and *GAPDH* Cq values were analyzed and compared between all sample groups and post-mortem intervals (PMI).

**Results:**

RIN values in MIA samples were significantly higher than those in CA samples. *GAPDH* was expressed significantly higher in MIA samples than in CA samples and 530 bp PCR products could be measured in all cases. *GAPDH* expression was significantly lower in samples with PMI >15 hours. As expected, the samples of the fresh frozen reference standard performed best in all analyses.

**Conclusion:**

MIA samples showed better RNA quality than CA samples, probably due to shorter PMI. Both had lower RNA quality and expression levels than fresh frozen tissue, however, remaining *GAPDH* RNA was still sufficiently intact. Therefore, other highly expressed genes are most likely also detectable. Gene array analysis should be performed to gain insight into the quality of entire post-mortem genomes. Reducing PMI will further improve the feasibility of demanding molecular research on post-mortem tissues, this is most likely more feasible with MIA than CA.

## Introduction

In most cancer patients, only tissues from the primary tumors are biopsied or resected for diagnostic and therapeutic purposes. Outside the context of studies clinicians do not biopsy recurrent or metastatic disease, unless it has therapeutic significance. This hampers the molecular comparison of primary and metastatic disease, despite the fact that it is now evident that there is not just intra-tumor heterogeneity [1], [2] but that there are also considerable molecular differences between primary tumors and metastases [3]–[6]. Chemotherapeutic and other systemic treatments based on genetic characteristics of the primary tumor may not work effectively on metastases due to changes of molecular targets, such as receptor conversion [7]–[9]. Knowing the molecular characteristics of metastases may help to target them specifically.

It is, therefore, necessary to pursue molecular research, comparing primary tumors and metastases. Post-mortem investigation is an opportunity to obtain tissue samples from (recurred) primary tumors and metastases for comparative molecular studies [10], [11]. The so-called “rapid autopsy” is performed soon after death, in order to minimize post-mortem degradation of collected tumor samples and allows for procurement of among others high quality RNA [12], [13].

Unfortunately, autopsies are rarely performed on patients who died of cancer. Bereaved relatives are often not willing to give their consent for conventional autopsy (CA), mainly because they feel that their loved one has suffered enough from the disease and they consider (further) mutilation of the deceased's body undesirable [14]–[16]. Minimally invasive autopsy (MIA) may be an acceptable alternative to CA [17], because with MIA, the body is imaged by CT and MRI and tissue samples from a (recurred) primary tumor and metastases are obtained through CT-guided biopsies, leaving the body intact.

Here we have investigated RNA in such biopsies as a measure of overall quality of the tissue for molecular studies. We studied whether the biopsies yielded: a) a sufficient amount of RNA and b) RNA of sufficient quality for downstream RNA analysis. By using RNA isolated from MIA, CA and fresh frozen ex vivo tissue in a RT-qPCR amplicon size assay, we were able to determine the levels of post-mortem RNA degradation and quality.

## Materials and Methods

In this prospective study RNA quality of post-mortem tissues was examined and compared to fresh frozen samples. The post-mortem tissues were obtained from two types of post-mortem examination: MIA and CA. Heart, kidney and liver tissue samples were collected at both MIA and subsequent CA in the same case, thereby excluding inter-patient variation between the two types of post-mortem samples.

Fresh frozen samples of the same three organ types, which had been obtained from living subjects, were culled from our frozen tissue bank. The three tissue types were selected based on accessibility during MIA, different rates of postmortem autolysis, availability in the frozen tissue bank and previous studies, showing acceptable results for basic molecular research with these tissue types [18], [19]. The collected tissue samples were not always free from pathological changes.

### Subject inclusion and clinical states

This study was approved by the Erasmus MC Medical Ethical Committee (file MEC-2011-055-amendment 002). All cases of in-hospital deceased adult patients whose bereaved relatives have given signed informed consent for both MIA and CA could potentially be included in this study protocol. Samples of fresh frozen residual tissue of heart, kidney and liver derived from surgical specimens or biopsies were provided by the Erasmus MC Tissue Bank and used according to the Dutch Code of Conduct 2011.

The autopsy samples were collected in the period between 11-28-2012 and 11-27-2013 whenever the responsible researcher (AvdL) was available for tissue sampling.

The time of death, entered by the subject's physician, and the time of tissue sampling at MIA and CA were registered. The time elapsed between death and the freezing of the sampled tissue was defined as the post-mortem interval (PMI). PMI is an important parameter known to influence tissue degradation [20] and thus RNA integrity, also taken into account in forensic pathology [21]. The MIA was always performed at the evening before the CA, therefore the PMI was longer in tissues collected at CA. Medical data from the last phase of life (fever, hypoxia, hypertension and diabetes) and patient body mass index (BMI) were obtained from the subject's medical records and the autopsy forms filled in by the subject's physician.

### Tissue sampling

During the MIA tissue samples were obtained with CT guided biopsies, using a 12-gauge needle. Immediately after each biopsy the sampled tissue was snap frozen in a 50 ml tube filled with pre-cooled isopentane on dry ice [22]. The samples were then placed in a pre-cooled aluminum vial, stored temporarily in a −80°C freezer and finally transferred into liquid nitrogen storage.

Immediately after MIA the body was returned to the mortuary with an ambient temperature of 4°C. The following day tissue samples of approximately 0.5 cm^3^ were collected from the same organs in the same subject during CA. Due to logistics at CA, snap freezing immediately after harvesting was not possible in all cases. In two cases the tissue samples were temporarily stored at 4°C before snap freezing. All collected samples were eventually stored in liquid nitrogen until RNA could be isolated.

Fresh frozen tissues were either derived from surgically resected tissues or from biopsies (heart). These tissue samples were stored in liquid nitrogen in the Erasmus MC Tissue Bank after being snap frozen using pre-cooled isopentane and liquid nitrogen.

Two extra heart samples (1 from MIA, 1 fresh frozen from the Erasmus MC Tissue Bank) were collected for training purposes. To prevent selection bias, these extra samples were both included in the analyses.

### RNA extraction, RIN measurement and frozen H&E sections

Depending on the size of the sample, 10 to 20 10 µm thick frozen sections were cut on a cryostat microtome (Microm HM560, Adamas, The Netherlands). The sections were transferred to 700 µL Qiazol (Qiagen, Hilden, Germany) and RNA was extracted from these sections using the miRNeasy kit (Qiagen, Hilden, Germany) according to the manufacturer's protocol. RNA was eluted in 40 µL RNase-free water and 1 µL of RNase inhibitor (20 U/µL; AB, California, USA) was added to avoid RNA degradation during further handling.

The RNA integrity number (RIN value) [23] of the extracted RNA and the RNA concentration (ng/µl) was assessed by on-chip-electrophoresis, using the BioAnalyzer (Agilent RNA 6000 Nano kit and BioAnalyzer 2100 Expert, Agilent Technologies, USA).

5 µm sections were cut and stained with Haematoxylin and Eosin (H&E) for morphological investigation of the samples used for RNA isolation. The morphologic tissue quality assessment comprised of 1) the representativeness of the sample (yes or no); 2) the presence of necrosis (yes or no); 3) scoring the degree of autolysis: no (morphology unaffected), moderate (mild loss of staining of nuclei; some detachment of endothelium in vessels), or severe (severe/complete loss of staining of nuclei; complete detachment of endothelium in vessels).

5 fresh frozen samples and 9 MIA samples contained so little tissue, that it was all needed for the RNA isolation and no histologic slide could be made. For 3 fresh frozen samples, 2 MIA samples and 6 CA samples the necrosis could not be scored, due to freezing artifacts rendering the interpretation of the morphology uncertain. These cases were registered as ‘no scoring possible’ (n/s), due to severe autolysis.

### cDNA synthesis and RT-qPCR

Some smaller tissue samples did not yield the required concentration of 100 ng/µl RNA for cDNA synthesis. In order to achieve a concentration of 100 ng/µl total RNA per sample, a volume containing 1 µg of RNA from all samples (to assure equal treatment of all samples) was transferred to another test tube and air dried by Vacuspin (SpeedVac AES1010, Savant, USA) for 30 minutes at 45°C. 10 µl of RNase free water was then added to reconstitute the RNA samples to the required 100 ng/µl total RNA.

cDNA synthesis of all samples, including a positive control (MCF7 cell line RNA) and a no template control (NTC), was performed as previously described [5].

Three pools of randomly picked cDNA samples were made by taking 1 µl of cDNA of 10 fresh frozen (pool 1), 10 MIA (pool 2) and 10 CA (pool 3) samples and diluting them twenty times in water. A fourfold dilution series was made of the initial pool samples to create a sensitivity curve. This additional step, consisting of fifteen samples in total, was added to each PCR assay to assess the efficiency of the assays.

To determine the quantity (degradation in post-mortem samples compared to the fresh frozen samples) and quality (acceptable length of RNA fragments for demanding downstream RNA based genomic techniques), quantitative real time polymerase chain reaction (RT-qPCR) with the *GAPDH* amplicon size assay (Table 1), as previously described by Viertler et al [24], was performed on all patient and control samples.

**Table 1:** GAPDH primer sequences and base pair lengths used for RT-qPCR analyses.

Primer	Primer sequence (5′ –> 3′)	Amplicon size	PCR efficiency
		base pairs	POWER
			(10(-1/slope))*
*GAPDH* common fwd	CCA CAT CGC TCA GAC ACC AT		
*GAPDH* rev	ACC AGG CGC CCA ATA CG	71	1,94
*GAPDH* rev	GTA AAC CAT GTA GTT GAG GTC	153	1,93
*GAPDH* rev	TTG ACG GTG CCA TGG AAT TT	200	1,86
*GAPDH* rev	ACT TGA TTT TGG AGG GAT CT	277	1,81
*GAPDH* rev	AAG ACG CCA GTG GAC TCC A	323	1,82
*GAPDH* rev	ACG ATA CCA AAG TTG TCA TG	530	1,71

* based on a 4 fold dilution series of pooled fresh frozen tissue derived cDNA samples.

### Data analysis

Differences in RIN values and Cq values, with respect to tissue types (heart, kidney and liver); sample types (fresh frozen, MIA and CA); PMI; clinical/patient related data and morphology scores were analyzed in SPSS (IBM SPSS Statistics, version 21.0) using the Mann-Whitney U test and the Paired Kruskal-Wallis test.

Since the Cq values were measured for 6 *GAPDH* base pairs per tissue sample, multiple testing needed to be applied [25], [26]. Therefore, the usual significance level of P = 0.05 was divided by 6, resulting in a significance level of P = 0.00833, which was used for the analyses of RT-qPCR outcomes.

## Results

### Sample collection

A total of 218 tissue samples from three different tissue types were collected. In 23 out of 24 autopsy cases the MIA and CA samples were collected from the same corpse. In 1 autopsy case, the samples had only been collected during MIA and not during the CA; therefore CA samples from another autopsy case were collected from the Erasmus MC Tissue Bank. For training purposes two additional heart samples were collected, one fresh frozen sample and one MIA sample. These were both included in the analyses.

All fresh frozen tissue samples were culled from the Erasmus MC Tissue Bank. Per sample type three tissue types were collected (see Table 2).

**Table 2:** Number of tissue samples per sample type per tissue type.

	Sample type				
		Fresh Frozen	MIA	CA	Total
**Tissue type**	**Heart**	25*	25*	24	74
	**Kidney**	24	24	24	72
	**Liver**	24	24	24	72
	**Total**	73	73	72	218

*extra samples collected.

The collected tissues were categorized based on post-mortem interval (PMI). The post-mortem intervals of our cases ranged from 10 to 59 hours. Six PMI categories were created, starting with the first category of up to 12 hours, each following category consisting of a post-mortem interval of 10 hours, and a last category consisting of 14 hours. An overview of the distribution of PMI categories for the included cases is given in table 3.

**Table 3:** Distribution of tissue samples per sample type over post-mortem intervals.

		Sample type			
		Fresh frozen	MIA	CA	Total
**PMI**	**0 h**	73	-	-	73
	**≤12 h**	-	12	-	12
	**13 h–24 h**	-	21	6	27
	**25 h–34 h**	-	31	18	49
	**35 h–44 h**	-	6	36	42
	**≥45 h**	-	3	12	15
	**Total**	73	73	72	218

An overview of the available (and potentially relevant) clinical variables is shown in table 4 and an overview of the morphologic tissue quality assessment of the samples is shown in table 5.

**Table 4:** Available patient conditions (in agonal phase) that could influence RNA quality.

		MIA and CA
**Condition**	**Fever**	40%
	**Hypoxia**	57%
	**BMI >25**	60%
	**Hypertension**	21%
	**Diabetes**	17%
	**Dyslipidemia**	8%

**Table 5:** Morphologic tissue quality assessment per sample type.

		Sample type		
		Fresh frozen	MIA	CA
		n = 73	n = 73	n = 72
**Representative**	**yes**	88%	88%	100%
	**no**	5%	0%	0%
**No H&E-slide** *		7%	12%	0%
		**n = 68**	**n = 64**	**n = 72**
**Autolysis**	**severe**	3%	11%	21%
	**moderate**	16%	50%	44%
	**no**	81%	39%	35%
**Necrosis**	**yes**	3%	3%	6%
	**no**	93%	94%	86%
	**n/s** **	4%	3%	8%

*No H&E-slide, all tissue used for RNA isolation.

**n/s  =  no scoring possible.

### RNA integrity

RIN values were established for 199 samples. In 6 fresh frozen samples, 3 MIA samples and 2 CA samples the RNA integrity could not be established, because the RNA yield of the sample was too low. These samples were excluded from the analyses, as not being able to establish RIN values was due to the size of the tissue samples and not to the tissue quality.

In 4 MIA samples and 4 CA samples the RNA integrity could not be established, due to extensive RNA degradation (RIN <1.0). These samples were included in the analyses with an RIN-value of 0 (zero), resulting in a total of 207 samples for the analysis.

**Figure 1:**
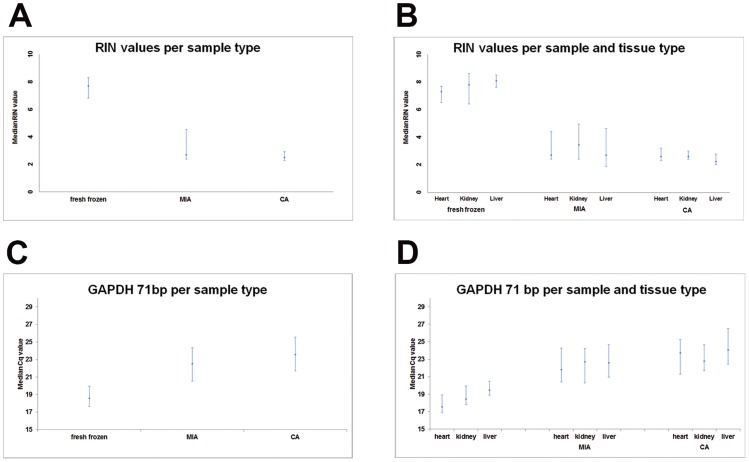
RNA integrity and GAPDH expression in fresh frozen, MIA and CA samples. Fig. 1A shows median RIN values (X-axis) of fresh frozen, MIA and CA samples (Y-axis). The fresh frozen samples, taken from surgical specimens, yielded high quality RNA. Both types of post mortem samples MIA and CA yielded RNA of lower quality. In Fig. 1B the samples are divided into 3 tissue types; heart, kidney and liver respectively. In Figs. 1C and 1D RT-qPCR results of the same samples are shown, in the same order. Since all GAPDH RT-qPCR assays in the 6-amplicon size assay showed similar results, only the results of the 71 bp assay are depicted here. The Cq value (Y-axis) obtained in fresh frozen samples is low, i.e. the GAPDH expression level is high. In the post mortem samples MIA and CA, GAPDH RNA is partly degenerated, resulting in higher Cq values corresponding with lower GAPDH expression levels.

The median RIN value and interquartile range for all three sample types are shown in Fig. 1A. The RIN values in fresh frozen samples were significantly higher than those in post-mortem samples (P<0.001, Unpaired Mann-Whitney test, significance level 0.05), and the RIN values in MIA samples were higher than those in CA samples (P = 0.032, Unpaired Mann-Whitney test, significance level 0.05).

Fig. 1B shows the median RIN values and interquartile range per sample type and tissue type. For each tissue type the RIN values of fresh frozen samples were significantly higher than those in post-mortem samples (P<0.001, unpaired Mann-Whitney test, significance level 0.05). No significant differences in RIN value per tissue type were found between MIA and CA. The morphological factor autolysis adversely influenced RNA integrity (P<0.001). Of the patient related agonal factors, hypoxia, BMI, hypertension and dyslipidemia did not influence RNA integrity, but fever and diabetes did have an adverse effect on the RIN value in post-mortem samples (both P = 0.006, unpaired Mann-Whitney test, significance level 0.05).

### RT-qPCR

RT-qPCR was performed on all 218 samples and Cq values could be established for 216 samples. In one case the RNA yield was too low, in the other case the Cq value could not be measured due to technical failure. These samples were excluded from the analyses concerning Cq values. Fig. 1C shows that median Cq values are lowest in fresh frozen samples for the *GAPDH* 71 bp assay. The Cq values in CA samples are highest. Fig. 1D show the Cq values per tissue type. Liver tissues have the highest Cq values for all sample types.

**Figure 2:**
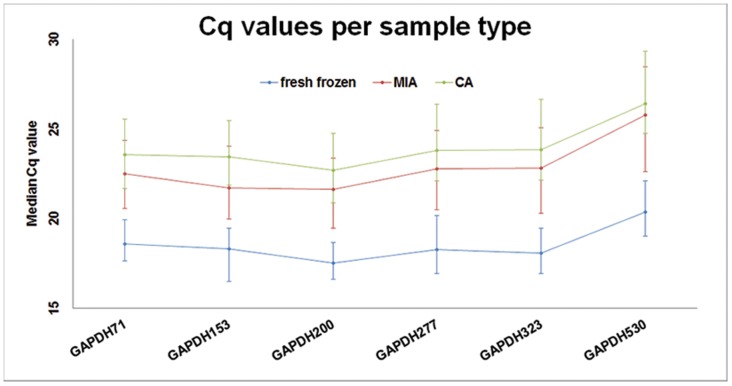
GAPDH size assay in fresh frozen, MIA and CA samples. The median Cq values (X-axis) of RNA derived from fresh frozen, MIA and CA samples obtained by RT-qPCR with various PCR product lengths (Y-axis) are shown. The fresh frozen samples always show lower Cq values, whereas the MIA samples show intermediate and the CA samples show high Cq values. This indicates that RNA in MIA samples is less degraded than RNA in CA samples. The parallel increasing Cq values between GAPDH323 and GAPDH530 indicate suboptimal RT-qPCR performance, rather than decreased GAPDH expression levels.

Fig. 2 shows the Cq values of all *GAPDH* amplicon sizes per sample type. The difference in Cq values between fresh frozen and post-mortem samples (MIA and CA) is significant for all *GAPDH* amplicon sizes (P<0.001, Paired Kruskal-Wallis test, significance level 0,00833). The difference in Cq values between MIA and CA samples is not significant for all *GAPDH* amplicon sizes, as shown in table 6. When the Cq values were stratified per tissue type, there were no significant differences between MIA and CA samples.

**Table 6:** P-values comparing sample types per GAPDH size assay.

Sample type	*P*-value					
	71 bp	153 bp	200 bp	277 bp	323 bp	530 bp
**Fresh frozen vs. MIA**	<0.001	<0.001	<0.001	<0.001	<0.001	<0.001
**Fresh frozen vs. CA**	<0.001	<0.001	<0.001	<0.001	<0.001	<0.001
**MIA vs. CA**	0.009	0.003	0.005	0.009	0.003	0.016

*Kruskal Wallis, significance level 0,00833*.

**Figure 3:**
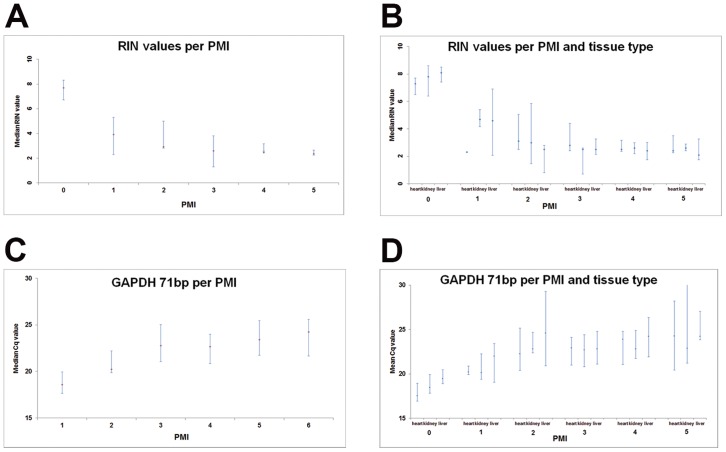
RNA integrity and GAPDH expression versus post-mortem intervals in MIA and CA samples. In Figs. 3A and B the median RIN value (X-axis) decreases with the post mortem interval (PMI; Y-axis). The fresh frozen specimens (PMI  = 0) show the highest RNA integrity. PMI 1 is exclusively comprised of MIA samples. PMI 2 to 5 are comprised of both MIA and CA samples. The actual PMI intervals are given in table 3. In Figs. 3C and D the results of RT-qPCR with GAPDH 71 bp are shown in the same context. Here we can appreciate that RT-qPCR is a more sensitive method of RNA integrity measurement, since it was possible to find significant differences not only between PMI 0 and PMI 1, as was also measured with RIN values, but also between PMI 1 and PMI 2.

The RNA integrity of all samples decreases with increasing post-mortem interval (see Fig. 3A). Fig. 3B shows the RIN values of the three different tissue types per PMI. The fresh frozen samples are represented by PMI category “0”. The RIN value in this category was significantly higher than in all other categories, but there were no significant differences in RIN values between PMI categories 1 to 5.

According to Fig. 3C, the *GAPDH* 71 bp assay Cq values increase with PMI (i.e. decrease of *GAPDH* expression level). Fig. 3D shows the GAPDH 71 bp assay Cq values per tissue type per PMI. Cq values in PMI category 2 are significantly higher than those in PMI category 1.

The morphological factors necrosis and autolysis had an adverse effect on *GAPDH* expression, with *P*-values ranging from <0.001 to 0.008 depending on amplicon sizes (results not shown). The *GAPDH* expression differed between patients who had dyslipidemia or a BMI higher than 25 and those without these symptoms (P ranges 0.001 to 0.008 and <0.001 to 0.015 depending on amplicon size), for patients with and without hypertension only the three longest GAPDH amplicons differed significantly (P<0.001, Paired Kruskal-Wallis test, significance level 0,00833), whereas hypoxia, fever and diabetes did not influence *GAPDH* expression.

## Discussion and Conclusion

RIN values were significantly higher in fresh frozen samples than in post-mortem samples. MIA samples showed higher RIN values than CA samples. Also, *GAPDH* expression was significantly higher in fresh frozen samples than in post-mortem samples. There were differences in *GAPDH* expression between MIA and CA samples, but they were significant for only 3 out of 6 *GAPDH* amplicon sizes. Since *GAPDH* amplicon sizes of up to 530 base pairs could be detected well within the limits of the assay's sensitivity, we conclude that the post-mortem samples still contained RNA of reasonable quality.

It is generally believed that post-mortem tissues with poor RNA integrity values (RIN value <5) are not suitable for molecular techniques [27]. In this study we showed that samples with low RIN values could still be used for determining gene expression with RT-qPCR.

The Affymetrix and Illumina gene array platforms respectively use 25-mer or 50-mer probes to detect gene expression. Since it is possible to amplify 530 bp *GAPDH* RNA fragments, this implies that detection of *GAPDH* with gene array technology must be possible. *GAPDH* expression in post-mortem tissue is 4 Cq, approximately 16-fold lower than in fresh frozen tissue (see Fig. 2). Since *GAPDH* is an abundantly expressed housekeeping gene (i.e. high copy number per cell), the transcript could still be measured in post-mortem tissue derived RNA with qPCR. Assuming post-mortem RNA degradation is a random process, all transcripts (i.e. the entire transcriptome) will be subjected to degradation to the same degree [28]. Therefore, less abundantly expressed genes (i.e. low copy number per cell) may become undetectable after this rate of degradation. Nonetheless, successful gene array analysis of post-mortem heart tissue was previously described [10]. Recently, Romero et al. published that even with low RNA quality RNA sequencing is possible, as long as the RIN values are accounted for during data analysis [29].

Handling and processing RNA may have major impact on RNA quality and therefore on the outcomes of the study. Inconsistency in executor and work performance, and contamination of RNA (with RNase or other genetic material) must be avoided when working with RNA [18], [30]. The experiments were therefore performed by one qualified and experienced researcher (AvL). To all RT-qPCR assays a dilution series was added to assess the performance of both the assay and the researcher's skills. The efficiency numbers (table 1) show that the RT-qPCR assays were all well performed, although the efficiency decreases with amplicon size. The observed (parallel) increase of Cq values in all samples (i.e. post-mortem as well as fresh frozen; Fig. 2) in relation to the increasing amplicon size may thus be due to decreasing PCR efficiency, rather than increased RNA degradation.

The integrity of post-mortem tissues is subject to various factors [20], [31]. Our results suggest that RNA in MIA tissue is less degraded than RNA derived from CA tissue. However, in this study CA was always performed after MIA, so the longer PMI is probably the explanation for this finding. Yet it is possible that differences intrinsic to MIA and CA play a role as well [9], [11], [32]. More extensive exposure of tissues to air, which occurs during conventional autopsies, could also have a negative influence on tissue quality. It is known that vacuum sealing of tissue specimens has a beneficial effect on RNA preservation [33].

Regarding patient conditions, our analyses show seemingly contradicting results: whereas hypoxia, BMI, hypertension, and dyslipidemia do not seem to have an effect on RIN values, hypoxia, fever and diabetes did not show significant differences between Cq values. The RNA quality according to the RIN values was significantly lower in cases with reported fever and diabetes, the RNA quality according to the Cq values was significantly lower in cases with dyslipidemia and a Body Mass Index (BMI) of >25, and in cases with hypertension, the Cq values differed significantly for only some GAPDH base pair lengths. The inconsistent results are probably due to the low number of samples from different cases.

Agonal factors, such as hypoxia and fever are known to have a negative influence on RNA quality and can lead to inhomogeneous RNA samples in different tissue types [34]. To our knowledge, the individual influence of hypertension, dyslipidemia and diabetes mellitus on post mortem tissue quality is unknown. Together however, these factors are described as “the metabolic syndrome”, which is associated with obesity [35]. The few differences found in RNA quality might therefore be explained by the confounder obesity. Obesity is in fact correlated with post mortem tissue quality, as it causes temperature insulation by fatty tissue, leading to higher core temperatures that are maintained longer. These higher temperatures enhance decomposition (autolysis and putrefaction) [36] or “warm ischemia” and cause faster degradation of RNA. Since the surrounding temperatures of both the hospital and mortuary have been similar for all cases in our study, they probably hardly affected the course of decreasing core temperatures, especially not to the extent obesity did.

According to Byard et al., obesity also complicates the autopsy, both in a practical manner and, together with the metabolic syndrome, in the diagnostic process [36]. In our experience, the combination of imaging and CT guided tissue biopsies eased these practical issues, and the tissue quality of MIA samples at least equals that of CA samples. On top of that, MIA could also be less expensive than CA [17]. The diagnostic aspects, however, remain to be further investigated.

In conclusion, although RNA integrity is lower in MIA and CA samples than in fresh frozen tissues, MIA and CA samples can be used to detect *GAPDH* PCR products up to 530 base pairs. This implies that tissue obtained by MIA yields a sufficient amount of RNA with a sufficient quality for gene array based research. Therefore, the MIA procedure is a feasible method for researchers to obtain metastatic tumor tissue for molecular translational research.

Potential advantages of MIA over CA for obtaining metastatic tumor tissue are the higher chance of getting consent from bereaved relatives, and the better feasibility to reduce PMI, which is the most crucial factor for high quality post mortem tissue for molecular analyses.
