# Correction: Post-Mortem Tissue Biopsies Obtained at Minimally Invasive Autopsy: An RNA-Quality Analysis

**DOI:** 10.1371/journal.pone.0118969

**Published:** 2015-03-05

**Authors:** The PLOS ONE Staff

Dr. Jan L. Robertus is not included in the author byline. He should be listed as the fifth author and affiliated with Department of Pathology, Erasmus MC, Rotterdam, The Netherlands. The contributions of this author are as follows: Conceived and designed the experiments, analyzed the data, contributed reagents/materials/analysis tools.

The correct citation is as follows: van der Linden A, Blokker BM, Kap M, Weustink AC, Robertus JL, Riegman PHJ, et al. (2014) Post-Mortem Tissue Biopsies Obtained at Minimally Invasive Autopsy: An RNA-Quality Analysis. PLoS ONE 9(12): e115675. doi:10.1371/journal.pone.0115675
